# Agile Transformation: How Employees Experience and Cope with Transformative Change

**DOI:** 10.1007/978-3-030-58858-8_16

**Published:** 2020-08-18

**Authors:** Dina Koutsikouri, Sabine Madsen, Nataliya Berbyuk Lindström

**Affiliations:** 6 IT University of Copenhagen, Copenhagen, Denmark; 7 University of British Columbia, Vancouver, BC Canada; 8 Department of Applied IT, University of Gothenburg, Gothenburg, Sweden; 9 Department of Politics and Society, Aalborg University, Aalborg, Denmark

**Keywords:** Agile transformation, Software development, Case study, Manufacturing

## Abstract

Modern manufacturing is highly competitive, requiring that organizations reduce lead times and achieve greater organizational flexibility, for example by implementing agile ways of working. However, studies show that incumbent firms have persistent problems with adopting and scaling such practices. In this paper, we present an empirical account of agile transformation in a large manufacturing company that has adopted the SAFe framework. Based on interviews, focus groups, and observation data, we identify three themes for understanding how employees experience and cope with transformative change by: 1) making sense of the new, 2) practicing with peers and 3) letting go of legacy. Key findings are that initially employees are more concerned with making sense of the new rather than with the implementation of agile itself and that implementation of agile happens very gradually over time rather than through major breakthroughs. Thus, it takes time for employees to weather change, become acquainted with the new way of working and stabilize how they work together in the agile teams and across the ARTs (Agile Release Trains). We contribute to extant literature with insight into the human implications of agile transformation.

## Introduction

Recently, there has been an outpouring of literature that seeks to explain why organizations should strive for agility to be able to respond quickly to change [1]. In larger organizational settings, becoming agile often requires the organization to undergo an agile transformation. Agile transformation refers to how large incumbent organizations change from their existing operating model to an agile way of working. This is accomplished through the adoption of principles, methods and frameworks that facilitate the scaling of agile development [2]. However, agile transformation and agile scaling are considered challenging because they require that employees in an organization change how they think, work and interact [2, 3]. Thus, as organizations attempt to become agile, employees face the challenge of letting go of traditional ways of working and embracing the ‘new’ thereby making agile transformation primarily a ‘people transformation’ [4]. Yet, main lessons from scholarly research are mostly presented from an *instrumental and managerial view* in terms of identifying, classifying, and mapping solutions onto transition challenges.

In this paper, we look at agile transformation from *the employees’ lifeworld perspective* to better understand the effort and agency of employees when their organizations undergo transformative change, for various reasons. For us as researchers, it is *both* a value position to emphasize employee agency rather than managerial drive *and* an avenue for shedding light on what management-initiated transformation ‘feels like’. Thus, the aim is to contribute to the emerging research agenda that focuses on the social aspects and human implications of large-scale agile transformations. To this end, we ask: *How do employees experience and cope with transformative change?*

We address this research question using a case study design [5], where we followed the agile transformation for one year by conducting interviews, focus groups and observations in a large Swedish manufacturing company. Thus, we have studied the employees experience of having to adopt the Scaled Agile Framework (SAFe) [2] and to adapt to a new operating model that emphasizes team interaction and agile roles rather than hierarchical power and traditional job titles. The paper advances current knowledge by focusing on agile transformation as a change process, which causes intense experiences that unfold over time as the employees make sense of the change and gradually adjust the way they work, and especially how they work together.

## Background

Agile transformation and scaling are challenging, because they require transformative change as well as figuring out how to make agile work outside the small-team context, which it was intended for and where it has proven successful [6]. Therefore, researchers as well as practitioners have also demonstrated a significant interest in understanding agile transformation and in supporting agile scaling [e.g. 3, 7–9].

Several frameworks have been proposed for scaling agile in larger organizational settings. The Scaled Agile Framework (SAFe) [2] is the most adopted model for scaling agile across the enterprise. However, there is scarce empirical evidence on how the SAFe framework is deployed (or to what extent it can be fruitfully implemented in a large distributed environment). Further, the existing literature indicates that reaping the benefits of agile principles at a large scale is inherently difficult [3, 8, 9]. Although this do not seem to deter organizations from implementing agile scaling efforts [see, e.g. 7] understanding the contingencies surrounding agile transformation appear more important than ever [10].

Establishing an agile development approach often requires transformation, but many organizations underestimate the efforts required to institute new ways of working [11]. In a recent review of 13 agile transformation cases [9], the authors identified nine key challenges associated with implementing agile methods on a large-scale, including: difficulty in defining concepts and terms, comparing and contrasting frameworks, readiness and appetite for change, top-down vs bottom-up implementation, overemphasis on 100% adherence over value, lack of evidence-based use, balancing organizational structure while adhering to large-scale methods, lack of evidence-based use, maintaining developer autonomy, and misalignment between customer and processes frameworks. They also note that since many problems are ‘subtle and can exist under the radar’ it is difficult to address all of them. Moreover, they urge researchers to move toward developing theory that captures the dynamic nature of transformation processes and evolution over time.

Paasivaara et al. [8] propose four lessons learnt for large-scale agile transformations: 1) consider using an experimental approach to transformation, 2) consider implementing the transformation step-wise in complex large-scale settings, 3) team inter-changeability can be limited in a complex large-scale product—specialization might be needed, and 4) not using a common agile framework for the whole organization, in combination with insufficient common trainings and coaching may lead to a lack of common direction in the agile implementation. Further, according to the The state of Agile Survey [12] ‘internal culture’ remains an obstacle to adopting and scaling agile practices successfully in many organizations.

In general, the growing body of literature shows consensus on factors that enable and hinder adoption of agile methods and the challenges of scaling agile practices. However, literature reviews show a lack of systematically conducted studies on large software development organizations adopting agile methods [11, 13]. Given the nascent stage of agile research and theory there is a strong call from the research community for more empirical studies on agile transformation [3, 7, 8].

## Method

In this paper, we investigate the experience of transformative change, while it is happening. Thus, our study follows the case of an ART (Agile Release Train) implementing the SAFe framework to change from waterfall to agile way of working. When we started collecting the data, the ART had entered the early stages of agile transformation. Data collection in the form of 25 interviews, two focus group sessions and several observations took place over a period of one year at the case company’s premises. Specifics regarding the case company have been anonymized for confidentiality reasons.

**Fig. 1.**
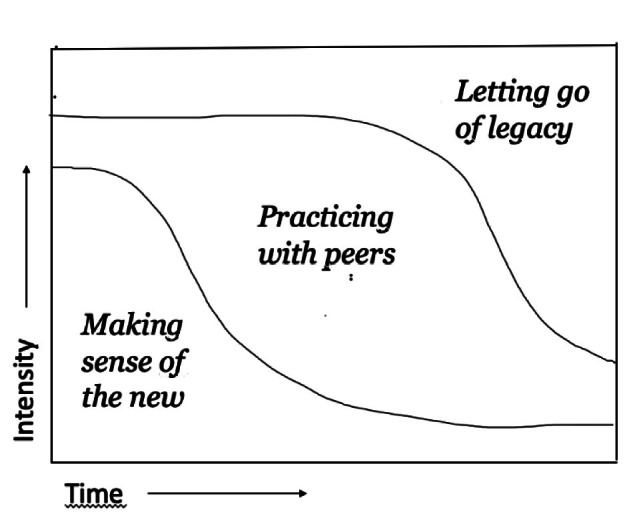
Three overlapping processes of coping with transformative change

The interviews lasted 60–90 min and where held with members of the ART, including roles such as systems developers, software testers, system architects, scrum masters, product owners, ART managers and agile coaches. Each of the focus group sessions lasted 90 min. The first session was carried out with four group managers and the second included eight scrum masters. To analyze the empirical data, we have applied Braun and Clarke’s [14] phases of thematic analysis. No a priori coding template was used as the purpose was to understand transformative change from the employees’ perspective rather than from a pre-existing theoretical point of view. In the first phase, we read the transcribed interviews and noted down ideas in a process of familiarization. Secondly, we conducted open coding to generate the initial codes. Next, the whole data set was grouped together under similar codes and then sorted into initial themes. In the third stage, we considered and conceptualized the themes in relationship to each other. As we examined how the practitioners experienced and were coping with the transition (from waterfall to agile) we began to realize that this emphasized three overlapping processes. We then refined and conceptualized these processes as three overlapping phases, which are illustrated in Fig. 1 (see the conclusion section).

## Case Study

The organization under study has realized that in order to stay relevant in a highly competitive marketplace, it must be able to respond quickly to change in customer demands and technology. Moreover, the organization’s competitiveness is increasingly relying on frequent releases of new/better software that improves the functionality of the physical products, rather than the physical products per se. Therefore, senior management has introduced agile methods and the SAFE framework as a solution that is intended to help the organization’s software development employees speed up their ability to release product embedded software often. However, our analysis shows that the shift from the plan-driven waterfall approach to delivery-oriented agile sprints is a major transformative change that involves many elements. These are listed below. The first two elements refer to top management decisions, while the latter three refer to aspects that our interviewees described as particularly challenging.

1. *Formal training*. Everybody is going through formal training.
2. *Co*-*located teams*. The previous departmental/functional area structure is replaced with a cross-functional team structure and if possible, team members are physically relocated to sit together in shared office space.
3. *Becoming a team*. The team members in the new teams have to get to know each other and figure out how to become a self-organizing team.
4. *Communication between teams*. The teams must find out if, when and how it is necessary to communicate between teams in the ART.
5. *Lag time*. Not everybody has switched to the agile way of working yet as it takes a long time for everybody to attend the basic formal training and to be allocated and physically moved into teams. Thus, employees who are trying to learn the new way of working must collaborate with employees, who are still working in the old way.

With the shift to agile nearly everything that the employees could previously take for granted is called into question, meaning that there is much cognitive and practical pressure to create new shared understandings about how to behave, work and relate to each other in the new organization.

### Making Sense of the New

The first process, making sense of the new, is characterized by the employees spending much mental energy on trying to understand the new agile working model. However, “…*it is hard, it is a totally different way of working and thinking…”* (Systems architect).

The employees understand the agile way of working by comparing it with what they are familiar with, namely the waterfall approach. In other words, the old is the frame of reference for making sense of the new. It is also clear from our data that an important step towards understanding the new is to prefer, and even glorify, the past. Thus, the new way of working is initially evaluated rather critically through professional and personal filters based on previous experience. Moreover, agile is subject to continuous individual and shared (re)interpretation and (re)negotiation.

To cope, the employees seek explanations and ask for facts and measurements. However, they still feel that they lack information. Moreover, they express that communication has become more burdensome after the change to agile, because the method prescribes that they must communicate more within the team, but they lack knowledge of who knows what and who can make which decisions.

*“I feel that I lack information. But I have been thinking about this a lot, and I cannot really say what I’m lacking…and that is quite confusing…I hear from a lot of people that we are missing information…but no one really knows what we are missing…I think it is because I have not adjusted…”* (Systems architect).

Several interviewees realize that these information and communication challenges are not the real problem, but rather a way of deflecting uncomfortable experiences associated with the change: *“We are still in the uncertainty and they are not really liking this”* (Scrum master). In general, our empirical data suggests that it is the individual’s intense experience of confusion, uncertainty and anxiety that carries and colours the sense making processes at the early stages of transformative change.

### Practicing with Peers

The second process, practicing with peers, foregrounds the employees’ efforts to, in their own terms, grow into the agile way of working at the coalface, because *“[t]he reality is that you cannot stop [development], because you want to learn how the team has to work together”* (Software developer). This is described positively, as the employees state that it is by trialing and learning agile together in the new teams, that they are able to collectively figure out what the concepts of agile, such as self-organizing teams, rapid feedback, prioritizing backlog and focusing on continuous learning, really mean for their day-to-day work. While the formal training is important for building basic understanding, it is by practicing with their peers that the employees start to adjust to the agile roles and work practices.

*“It is a new thing…so everybody is trying to learn…the Scrum master is new, so he needs to learn more, the Product Owner needs to learn about it, and of course all other parts of the organization…to also even communicate with each other.”* (Software Developer).

However, it is challenging and time-consuming to practice new relationship types and develop new interaction norms through socializing, while also attempting to do the actual work in another way, using a new language. Consequently, the interviewees experience that because so many things are new for so many people, there is a relatively long period with less productivity; despite the aim being to speed up software development.

*“It takes time, takes time…I just want to go to the same level as before agile. We had a better productivity than now. But I’m expecting it will happen. I’m hoping*.” (Software Tester).

The employees understand that the software development part of the organization is going through a major learning process. Hoping that people will learn and that things will get better in a foreseeable future seems to be the main coping mechanism. The future-orientation helps the employees deal with the experiences of productivity loss and collaboration challenges that seems to characterize the change at the middle stages.

### Letting Go of Legacy

Once the agile transformation had picked up pace, most employees sought routine in their daily operations and interactions, which contributed to a surge to make it work. Crucial to this ‘reorientation’ period, is the third process of letting go of legacy. However, some aspects are more difficult to let go of than others, particularly hierarchical structure, cultural values, and identity-defining skills.

*“What is hard at the moment, is that we are still living in the traditional project management world and this cannot be changed in an afternoon!”* (Product Owner).

Indeed, the interviewees’ hierarchical organizational structure difficult to let go of. This challenge is referred to as ‘adopting a new mindset’. The employees experience the new agile teams and roles as vastly different from how they used to work. In particular, the team members have to let go of having a boss that they can go to for help. Instead they have to embrace the freedom and responsibility of self-organizing teams. While some employees enjoy this, others find it uncomfortable and feel more alone with the burden of decision-making.

Moreover, some employees who previously had the title of project managers, now have to function as scrum masters. This is a difficult change as old conceptions of what it means to be a manager has to be unlearned in favor of a more facilitating approach. Reminding oneself not to fall back into old habits and encouraging co-workers to remember the new are important coping mechanisms:

*‘‘I find it difficult to avoid meddling with technical issues since in my old role as group manager I was responsible for the team and technical side. Now I have to let go of the technical responsibility to the product owner. I have to work hard to get myself into this new mindset.”* (Group manager).

For the individual employee, the change to agile creates an intense experience of anxiety about the relevance of one’s skills and ultimately, one’s relevance in the new organization. This is turn means that fear of letting go co-exists with a desire to be *as* or *more* productive than before the shift to agile. Therefore, the task is to keep doing the hard work of understanding agile, its feasibility and its desired outcome, while simultaneously hooking it into the prevailing work system, which is ultimately very difficult to discard.

## Conclusion

Our research is an attempt to draw on lifeworld interviews with employees to shed new light on why agile transformation presents challenges for established organizations.

In Fig. 1, we summarize our research findings. This process model delineates how the employees experience and cope with transformative change over time through three recursive processes. Thus, initially the employees are more concerned with making sense of the new by comparing it with the past than with the implementation of agile itself. This is a very intense experience propelled by anxiety and uncertainty. Next, an important way for the employees to understand what is required of them and their new roles, is to practice with peers, and despite a drop in productivity due to learning, to hope that things will get better in the foreseeable future. However, it is difficult to let go of the hierarchical structure and identity-defining values and skills and to begin to form a new mindset. Therefore, the implementation of the agile way of working happens very gradually through subtle shifts in meanings and practices rather than through major breakthroughs. At this latter stage, the letting go of legacy is driven forward by the employees’ desire for the change to be ‘over’ and for the new to work as ‘normal’.

Above all our study highlights that employees will take their time to cope with change, because it is a time- and energy consuming endeavor, both emotionally and practically as well as individually and collectively. We find it interesting that our findings led us to emphasize slowness as key to understanding and perhaps also overcoming some of the challenges of agile transformation. This is of course an uncomfortable insight, for the case company as well as in general, as agile transformation is undertaken to speed up software development and keep up with a competitive marketplace. Overall, the lessons from this case study are relevant for leaders of organizations contemplating large-scale agile transformation.

Future research will assist in determining the extent of generalizability to other organizational contexts facing transformative change. We believe that further research into the three empirically derived phases of change has potential to uncover how members of organisations collectively enact transformative change; thus, acknowledging and normalizing the silent individual struggles, the role of hope when the going gets tough as well as the effort and agency of employees in acquiring new skills and engaging with each other to socialize their way into the new way of working. It will be important to further investigate other types of emotive processes or mechanisms involved in dealing with similar types of transformative change efforts. We hope to have provided a start in this direction.
